# MYELOstudy: lymph node stage of aggressiveness in medullary thyroid cancer—a retrospective multi-center study analysis

**DOI:** 10.1007/s13304-025-02513-6

**Published:** 2026-03-06

**Authors:** Theodossis Papavramidis, Angeliki Chorti, Sohail Bakkar, Marco Raffaelli, Andro Košec, Van Trung Hoang, James Y. Lim, Volkan Genc, Michele N. Minuto, Pietro Giorgio Calò, Andrzej Hellmann, Giacomo Di Filippo, Lampros Karakozis, Alexandra Chrisoulidou, Viyey Kishore Doulatram Gamgaram, Chutintorn Sriphrapradang, Jean-Christophe Leclère, Aida Orois, Demarchi Marco, Muthuswamy Dhiwakar, Loredana de Pasquale, Antoine Buemi, Cédric Nesti, Rumen Pandev, Andres Chala, Selen Soylu Yalıman, Jiannis Hajiioannou, Shirley Yuk-Wah Liu, Sergii Cherenko, Nikolaos Voloudakis, Ioannis Koutelidakis, Konstantinos Nastos, Ramacciotti Constanza, Chiara Dobrinja, Maximilian Brunner, Lovenish Bains, Ramakanth Bhargav Panchangam, Fábio Muradás Girardi, Akif Enes Arikan, Hadj Omar El Malki, Michael de Cillia, Luigi Oragano, Ioannis Pliakos, Moysis Moysidis, Francesco Pennestrì, Carmela De Crea, Mateo Čukman, Andro Tarle, Olga S. Senashova, Maisie L. Shindo, İlgiz Tüzken, Mustafa Anil Turhan, Emanuela Varaldo, Manuela Albertelli, Fabio Medas, Gian Luigi Canu, Federico Cappellacci, Maciej Śledziński, Giovanni Lazzari, Eleonora Morelli, Stavros Karakozis, Maria Boudina, Michael Katsamakas, Marta Iturregui Guevara, Rangsima Aroonroch, Martí Manyalich, Oscar Vidal, Frederic Triponez, Nathalie Masse, Lokesh Kathirvel, Rajeshwari Muthusamy, Paula La Rubia, Luca Castellani, Burlacu Maria-Cristina, Furnica Raluca-Maria, Reto Kaderli, Serkan Teksoz, Tom Chi-Man Chow, Man Sze Lai, Liuchiia Shchekaturova, Evangelia Bellou, Maria Banus, Roxana Da Milano, Manuela Mastronardi, Robert Grützmann, Luiz Alberto Hauth, Aliende Lengler Abentroth, Onur Dulgeroglu, Cihan Uras, Giuseppe Placentino, Monica Leutner

**Affiliations:** 1 1st Propaedeutic Department of Surgery, AHEPA University Hospital, Aristotle University of Thessaloniki, Thessaloniki, Greece; 2 Department of Minimal Invasive Endocrine Surgery, Euromedica Kyanos Stavros, St. Kiriakidi 1, 54636 Thessaloniki, Greece; 3 Department of Surgery, Faculty of Medicine, the Hashemite University, Zarqa, 13131 Jordan; 4 Centro Di Ricerca in Chirurgia Delle Ghiandole Endocrine E Dell’Obesità, UOC Chirurgia Endocrina E Metabolica, Fondazione Policlinico Universitario Agostino Gemelli IRCCS, Università Cattolica del Sacro Cuore, Rome, Italy; 5 Department of Otorhinolaryngology and Head and Neck Surgery, University Hospital Center Sestre Milosrdnice, Zagreb, Croatia; 6 Department of Radiology, Thien Hanh Hospital, Buon Ma Thuot, Vietnam; 7 Endocrine Surgery, Division of Surgical Oncology, Thyroid and Parathyroid Center, OHSU, Portland, OR USA; 8 Department of Endocrine Surgery, Ankara University Medicine Faculty, Ankara, Turkey; 9 Department of Surgical Sciences (DISC), University of Genoa, Genoa, Italy; 10 Endocrine Surgery Unit, Polyclinic Hospital San Martino, Genoa, Italy; 11 Department of Surgical Sciences, University of Cagliari, Cagliari, Italy; 12 Division of General, Endocrine and Transplant Surgery, Medical University of Gdańsk, Gdańsk, Poland; 13 Endocrine Surgery Unit, Verona University Hospital, Verona, Italy; 14 Thyroid Surgery Clinic IKE, Athens, Greece; 15 Theageneio Cancer Hospital, Thessaloniki, Greece; 16 Regional University Hospital of Malaga, Malaga, Spain; 17 Department of Medicine, Faculty of Medicine Ramathibodi Hospital, Mahidol University, Bangkok, Thailand; 18 Head and Neck Surgery, Brest University Hospital, Brest, France; 19 Hospital Clinic, Barcelona, Spain; 20 Department of Thoracic and Endocrine Surgery and Faculty of Medicine, University Hospitals of Geneva, 4 Rue Gabrielle Perret-Gentil, 1211 Geneva, Switzerland; 21 Kovai Medical Center and Hospital, Coimbatore, India; 22 Thyroid Unit - ASST Santi Paolo e Carlo - San Paolo University Hospital, Milan, Italy; 23 Saint Luc Hospital, Brussels, Belgium; 24 Department of Visceral Surgery and Medicine, Inselspital, Bern University Hospital, University of Bern, Bern, Switzerland; 25 Medical University of Sofia, Sofia, Bulgaria; 26 Head and Neck Department Oncologos del Occidente, Universidad de Caldas, Manizales, Colombia; 27 Faculty of Medicine, Department of General Surgery-Endocrine Surgery Division, Istanbul University-Cerrahpaşa, Istanbul, Turkey; 28 Head and Neck Department, Faculty of Medicine, School of Health Sciences, University Hospital of Larissa, University of Thessaly, Larisa, Greece; 29 Department of Surgery, Alice Ho Miu Ling Nethersole Hospital & Prince of Wales Hospital, Chinese University of Hong Kong, Tai Po, Hong Kong; 30 International Medical Center “CITIDOCTOR”, Kiev, Ukraine; 31 Gennimatas General Hospital, Thessaloniki, Greece; 32 Evgenidio Hospital, Athens, Greece; 33 Department of Endocrinology, Private University Hospital of Cordoba, Cordoba, Argentina; 34 Department of Medical and Surgical Sciences, University of Trieste, Cattinara Teaching Hospital, Trieste, Italy; 35 University Hospital Erlangen, Erlangen, Germany; 36 Maulana Azad Medical College & Lok Nayak Hospital, New Delhi, India; 37 Endocrine and Metabolic Surgery, Bhargav Endocrine Hospital, Vijayawada, India; 38 Head and Neck Surgery Department, Hospital Ana Nery, Santa Cruz do Sul, Brazil; 39 Acibadem MAA University, Istanbul, Turkey; 40 Mohammed Vth University in Rabat, Morocco, Medical School, Surgery Department ‘A’ and Clinical Research and Epidemiological Laboratory, Ibn Sina Hospital Rabat, Rabat, Morocco; 41 Department of Surgery, Saint John of God Hospital, Salzburg, Austria; 42 San Biagio” Hospital - ASL VCO - Domodossola (VB), Genoa, Italy

**Keywords:** Medullary thyroid carcinoma (MTC), Aggressiveness, Thyroid cancer, Surgery, Surgical oncology

## Abstract

Medullary thyroid carcinoma (MTC) is a rare, but biologically aggressive cancer that accounts for 1–2% of all thyroid malignancies. Its aggressiveness has been linked to distinct clinicopathological features. Novel pathological predictors of aggressiveness have also been described in the literature. However, these remain contentious, to date. To assess the prognosticators of aggressiveness for MTC and establish potentially novel ones. The primary endpoint was to establish predictors for central and/or lateral cervical nodal metastatic disease. Whereas secondary endpoints include defining biochemical and histopathologic markers of aggressiveness. A multi-center retrospective analysis of prognosticators of aggressiveness in MTC. 785 patients with MTC were enrolled. The mean age was 56 years with a female to male ratio of 1: 1.7. Regression analysis demonstrated that aggressive prognosticator predictive of nodal metastasis included: age, pre- and post-operative Calcitonin and CEA levels, tumor size, multifocality, capsular and lymphovascular invasion, the presence of extrathyroidal extension, Ki-67, and RET positivity, desmoplasia, and inflammatory scores. This study provides an expanded spectrum of prognosticators of tumor aggressiveness. Furthermore, it also highlights the potential meaningful therapeutic implications of preoperative inflammatory scores, tumor desmoplastic reaction, and metastatic nodal ratio.

## Introduction

Medullary thyroid carcinoma (MTC), the thyroid tumor with amyloid, was first described in 1959. It is considered a thyroid tumor because of its anatomical location, however in reality it is a neuroendocrine tumor as it originates from parafollicular cells (C-cells) which are of neural crest origin. MTC is the third most common thyroid malignancy. According to the most recent surveillance, epidemiology, and end results (SEER) data, it accounts for 1–2% of all thyroid malignancies. Despite being relatively rare, it is associated with considerable morbidity and mortality, accounting for 13% of all deaths from thyroid cancer [1–4]. The majority of MTCs (75%) are sporadic, while approximatively 25% are hereditary. These can occur solely (familial MTC) or as part of multiple endocrine neoplasia (MEN) syndrome. The oncogenesis of hereditary MTC is related to a gain of function mutation in the RET proto-oncogene located on chromosome 10, and more than a hundred mutations have been described. The oncogenesis of sporadic tumors is similar to that of its hereditary counterpart in 50% of cases, and unknown in the remainder of cases. Exon 16 / M918T is the most common mutation [2, 3, 5].

Sporadic MTC typically occurs between the fourth and sixth decades of life. Prognosis depends on age at diagnosis and disease stage. Overall, the ten-year survival rate for sporadic MTC stages I, II, III, and IV are 100%, 93%, 71% and 21%, respectively [2].

Due to the MTCs embryogenic origin of C-cell they are unresponsive to radioactive iodine ablation (RIA) therapy, and surgery remains the mainstay management for these tumors. Therefore, a thorough preoperative assessment of these tumors is of utmost importance for adequate surgical planning. Calcitonin is not only a confirmatory test, but it also reflects tumor burden, guides preoperative diagnostic imaging, governs the extent of surgery required, and serves as a marker for disease recurrence. Its levels should be cautiously interpreted in the context of age, sex, and tumor differentiation. Factors that negatively influence calcitonin levels (falsely low) include female sex, tumor de-differentiation, and the “hook effect” related to assays in widely disseminated MTC. Whereas factors that augment calcitonin levels include multifocality and concurrent calcitonin-secreting pathology. However, in extremely rare cases MTC may be negative for calcitonin [2].

The prognosis of MTC is usually not as favorable as well differentiated thyroid cancers. However, if discovered early, appropriate surgery can be curative. The 10-year survival of MTC is generally between 45 and 85% [2]. The outlook for MTC depends on several factors. Negative (poor) prognosticators include high tumor grade (high mitotic activity), older age, male sex, sporadic forms, cervical nodal metastases and the absolute number of metastatic nodes, distant metastasis, the presence of capsular and/or lymphovascular invasion, extrathyroidal extension, calcitonin and CEA doubling times, and tumors with *RET* / m918T mutation[2, 4, 6–9]. Furthermore, tumour desmoplastic stroma (desmoplastic reaction) has been recently assumed as a negative prognosticator [10–13]. Moreover, nodal ratio rather than the absolute metastatic lymph node count and tumour inflammatory markers such as neutrophil-to-lymphocyte ratio (NLR), platelet-to-lymphocyte ratio (PLR), and lymphocyte-to-monocyte ratio (LMR) have been gaining grounds as prognosticators of aggressiveness in MTC[14–18]. Doubling times for postoperative serum calcitonin and CEA levels, defined as the interval of time in which the tumor markers levels double, are also established as prognostic factors[19]. Positive (favourable) prognosticators include young age, female sex, familial forms (patients with MEN 3 have a more aggressive clinical course than those with MEN 2), and microcarcinomas [20, 21].

The primary endpoint of this study was to assess the correlation between tumoral clinicopathological features and the presence of cervical nodal metastasis and its stage (i.e. central and/or lateral). Furthermore, several other parameters such as tumor stromal desmoplasia, lymphovascular infiltration, inflammation markers were evaluated as an independent risk factor for aggressive disease.

## Methods

With the aim of analyzing prognosticators of tumor aggressiveness in MTC, a retrospective study was conducted on a broad multi-center international scale. Forty-one centers from twenty-two countries around the globe participated in this study. The clinical charts of all adult MTC patients (older than 18 years), surgically managed between January 2013 and December 2023 were collected. Data collection included patients’ demographics, preoperative biochemical profiles, preoperative diagnostic imaging, the extent of surgery performed, detailed postoperative histopathology reports recording tumor size, pathologic features (capsular and/or lymphovascular invasion, multifocality, and extrathyroidal extension), grading features (mitotic count, fibrosis, and necrosis), tumor stromal desmoplasia and tumor-stroma ratio and cell type, staining for calcitonin, CEA, thyroglobulin, Ki-67, *RET*, *K-RAS*, *N-RAS*, *H-RAS*, CD 133, and CD 44. Detailed descriptive data regarding nodal status (number of lymph nodes harvested, number of metastatic lymph nodes, nodal ratio, size of metastatic deposits, and the presence/absence of extranodal extension) were also collected. Lastly, postoperative follow-up data collected included postoperative calcitonin and CEA kinetics, disease-free and overall survival rates.

As our study is a multicentre study with data being shared internationally, compliance with ethical standards was assured by applying a uniform data collection, analysis, and sharing scientific policy that ensures maintaining data confidentiality and its use for scientific purposes only. Furthermore, all procedures performed in this study were in accordance with the ethical standards of the institutional and/or national research committee and with the 1975 Helsinki declaration and its later amendments or comparable ethical standards. This research obtained the Institutional Review Board (IRB) approval of each participating institute. However, Patients’ informed consent was waived because of the retrospective nature of this study.

The study was registered in Clinicaltrials.gov (registration number: NCT06292988).

### Statistical analysis

Quantitative variables that follow a normal distribution were reported as means ± standard deviation or median (interquartile range) for those that don’t follow a normal distribution. Qualitative variables were reported as frequencies and relative frequencies. Sample normality was tested using the Kolmogorov–Smirnov test. All of the continuous variables didn’t follow a normal distribution, so non parametrical tests were used. To compare the median values of the quantitative variables in the categories of binary categorical variables, the Mann–Whitney test was used. While to compare the median values of the quantitative variables in the categorical variables with 3 categories or more, the Kruskal–Wallis test was used. The Bonferroni method was used to further compare the medians in the categories of the qualitative variable (with the appropriate correction applied). Finally, with the Spearman correlation index (non-normal distribution) and with the X^2^ test (between categorical variables), possible correlations and dependencies between the variables were investigated. The statistical processing of the sample was done using the statistical package “Statistical Package for the Social Sciences (SPSS)” version 27 (IBM), (SPSS Inc., Chicago, IL, USA). In all cases, a *p*-value < 0.05 was set as the level of statistical significance for a two-tailed test.

## Results

The data collected and the missing data are demonstrated in Table 1. The parameter that was excluded from further multifactorial analysis was the tumor-stromal ratio due to very high level of missing data (98.30%). Moreover, analysis taking into consideration the cell type of the tumor was also omitted due to the fact that most MCTs had as predominant type of cell the C-cells.

**Table 1:** Data collected per category

Variables	N (Total 785)	% missing values
Demographics		
Age [at the time of surgery]	779	0.80%
Sex	780	0.60%
BMI (kg/m2)	600	23.60%
Preoperative Data		
TSH (mIU/L)	595	24.20%
fT3 (pg/ml)	322	59.00%
fT4 (ng/ml)	424	46.00%
Calcitonin (pg/ml)	624	20.50%
CEA (ng/ml)	367	53.20%
PTH (pg/ml)	470	40.10%
Calcium (mg/dl)	575	26.80%
White Blood Cells (K/L)	546	30.40%
Neutrophils (/μL)	483	38.50%
Lymphocytes (/μL)	488	37.80%
Monocytes (/μL)	487	38.00%
Platelets (/μL)	549	30.10%
Mean Platelet Volume	349	55.50%
Platelet Distribution Width	152	80.60%
Number of Positive Lymph Nodes	640	18.50%
Distant metastasis	689	12.20%
Pathology report		
Type of surgery	777	1.00%
Number of lymph nodes resected	644	18.00%
Number of positive lymph nodes	288	63.31%
Tumor stage (TNM)	770	1.90%
Lymph node stage (TNM)	775	1.30%
Distant metastasis (TNM)	774	1.40%
Diameter of tumor (mm)	758	3.40%
Focality	764	2.70%
Extrathyroidal extension	766	2.40%
Capsular invasion	749	4.60%
Calcitonin Staining	635	19.10%
CEA Staining	339	56.80%
Thyroglobulin Staining	404	48.50%
RET staining	275	65.00%
HRAS Staining	126	83.90%
KRAS Staining	130	83.40%
NRAS Staining	128	83.70%
Ki-67 (> 5%)	248	68.40%
Mitotic count (> 5)	180	77.10%
CD133 Staining	91	88.40%
CD44 Staining	86	89.00%
Tumor necrosis	498	36.60%
% necrosis	113	85.60%
Lymphovascular invasion	664	15.40%
Fibrosis	501	36.20%
Cell types	471	40.00%
Desmoplasia	391	50.20%
Tumor-stroma ratio	13	98.30%
Tumor deposit	305	61.10%
Postoperative Follow-Up		
Calcitonin	648	17.50%
Post-op day of calcitonin measurement	642	18.20%
CEA	455	42.00%
Post-op day of CEA measurement	444	43.40%
Suspicious/positive lymph nodes	663	15.50%
Aggressiveness Metrics		
Calcitonin doubling time (months)	150	80.90%
CEA doubling time (months)	114	85.50%
Structural doubling time (months)	215	72.60%
Death	671	14.50%
Survival (months)	519	33.90%
Extra Variables		
Neutrophil-to-lymphocyte ratio	313	60.13%
Platelet-to-lymphocyte ratio	488	37.83%
Lymphocyte-to-monocyte ratio	487	37.96%
Lymph node ratio	287	63.40%

### Demographic characteristics of MTCs

The study cohort included 785 patients. The mean age of the patients was 56 years. There were 493 females, with a male to female ratio of 1:1.71. Table 2 gives a detailed report of the studied demographics.

**Table 2:** Demographics and preoperative biochemical and haematological data of the patients

Variables	Median (Interquartile range)
Age [at the time of surgery]	56 (22)
BMI (kg/m2)	25.2 (5.9)

	Number of patients (%)
*Sex*	
Male	287 (37%)
Female	493 (63%)

Preoperative Data	
Variables	Median (Interquartile range)
TSH (mIU/L)	1.8 (1.8)
fT3 (pg/ml)	2.6 (1.6)
fT4 (ng/ml)	0.3 (0.9)
Calcitonin (pg/ml)	144.5 (922.7)
CEA (ng/ml)	12.4 (54.7)
PTH (pg/ml)	47.2 (32.5)
Calcium (mg/dl)	9.3 (1.1)
White Blood Cells (K/L)	6945 (2700)
Neutrophils (/μL)	4260 (2220)
Lymphocytes (/μL)	2030 (970)
Monocytes (/μL)	360 (475)
Platelets (/μL)	246,000 (92,000)
Mean Platelet Volume	9.7 (2.3)
Platelet Distribution Width	15.9 (34.7)
Neutrophil-to-lymphocyte ratio	2.08 (1.28)
Platelet-to-lymphocyte ratio	121.47 (65.21)
Lymphocyte-to-monocyte ratio	6.43 (28.91)

	Number of patients (%)
*Distant metastasis*	
Yes	27 (4%)
No	662 (96%)

### Preoperative characteristics of MTCs

The majority of patients were euthyroid and had normal serum biochemical and hematologic profile. The pathological biochemical values corresponded (as expected) to calcitonin and CEA levels. The mean basal calcitonin level was 144.5 pg/ml and the mean CEA level was 12.4 ng/ml. Table 2 depicts the above mention data in details. In the above-mentioned table, there are also “new variables” that could act as prognosticators of aggressivity such as neutrophil-to lymphocyte ratio, platelet-to-lymphocyte ratio and lymphocyte-to-monocyte ratio.

### MTCs characteristics according to pathology reports

Pathology reports helped us determine the type of operation performed in each case. Predominantly, total thyroidectomy with central neck compartment neck dissection, therapeutic or prophylactic, was performed in 40.9% of cases. Sole total thyroidectomy was performed in 23.2% of the patients. Total thyroidectomy accompanied with central and single lateral compartment was performed in 20.5% of the patients. Finally, total thyroidectomy with central and both lateral compartments was performed in 15.4% of cases.

Concerning the characteristics of the tumor, its mean size was 15 mm (SD 18 mm), with 77.5% of the cases being unifocal. Capsular and lymphovascular invasions were reported in 19.2% and 27.3% of the cases respectively, while extrathyroidal extension was present in 14.5% of the cases. A mitotic count of > 5 was demonstrated in 37.8% of the cases and Ki-67 more than 5% was present in 36.7% of the cases. In small percentages, the tumor presented: (i) necrosis (13.9% of cases), (ii) fibrosis (16.6% of cases), (iii) deposits (11.1% of cases), and (iv) desmoplastic reaction (in 6.9% of cases).It is worth mentioning, the various predominant cellular populations in MTCs (C-cells in 67.3%, spindle cells in 10%, plasmacytoid in 5.3%, squamous in 2.3%, clear-cells in 2.1%, giant-cells in 1.7%, oncocytes in 1.7%, angiosarcoma-like cells in 1.1%, melanotic cells in 0.8%, amphicrine cells in 0.8%). Finally, Table 3 depicts the various stainings of MTCs. As expected, there is intense staining for calcitonin and CEA, while there is negative staining for thyroglobulin. CD133 and CD 44 stainings show positivity in half of the cases. Gene sequencing for *RET, H-RAS, K-RAS, and N-RAS* was positive in 21.5%, 3.2%, 3.8%, and 1.6% of cases.

**Table 3:** TNM staging and staining and genetic characteristics of the studied MTCs

Variables		Number of patients (%)
Tumor stage (TNM)	Τ1	473 (61.4%)
	Τ2	164 (21.3%)
	Τ3	100 (13%)
	Τ4	30 (3.9%)
	ΤΧ	3 (0.4%)
Lymph node stage (TNM)	Ν0	416 (53.7%)
	Ν1a	169 (21.8%)
	Ν1b	132 (17%)
	ΝΧ	58 (7.5%)
Distant metastasis (TNM)	Μ0	712 (92%)
	Μ1	35 (4.5%)
	ΜΧ	27 (3.5%)

Variables		Number of patients (%)
Calcitonin staining	Negative	11 (1.7%)
	Mild	152 (23.9%)
	Moderate	71 (11.3%)
	Intense	401 (63.1%)
CEA staining	Negative	46 (13.6%)
	Mild	85 (25.1%)
	Moderate	23 (6.8%)
	Intense	185 (54.6%)
Thyroglobulin staining	Negative	354 (87.6%)
	Mild	43 (10.6%)
	Moderate	2 (0.5%)
	Intense	5 (1.3%)
CD133 staining	Yes	47 (51.6%)
	No	44 (48.4%)
CD44 staining	Yes	46 (53.5%)
	No	40 (46.5%)
RET	Positive	59 (21.5%)
	Negative	216 (78.5%)
HRAS	Positive	4 (3.2%)
	Negative	122 (96.8%)
KRAS	Positive	5 (3.8%)
	Negative	125 (96.2%)
NRAS	Positive	2 (1.6%)
	Negative	126 (98.4%)

As far as lymph node data extracted from the pathology reports is concerned, one can see that the median number of lymph nodes resected was 23 with an interquartile range of 29 lymph nodes. Among these lymph nodes, the median number of positive lymph nodes was 6, with an interquartile range of 9 lymph nodes. The mean lymph node ratio (positive to negative lymph nodes) was 0.35 with an interquartile range of 0.88.

Finally, distant metastasis was present in 4.5% of the patients.

According to the American Joint Committee on cancer (AJCC) tumor, node, metastasis (TNM) staging, more than 55% of cases were T_1_N_0_M_0_. Table 3 depicts in detail the respective T, N and M stages in the studied cohort.

### Follow-up characteristics of MTCs

Calcitonin and CEA doubling times were 9 and 8 months, respectively.

Postoperative follow-up data demonstrated calcitonin and CEA normalization in 64% and 80% of patients, respectively. Measurements were obtained on 43 and 62 days after surgery, respectively. A postoperative metastatic lymphadenopathy was reported in 17.6% of cases. The overall survival rate of the study cohort was 93.9%, whereas the mean survival rate was 53 months. Table 4 explains in detail all postoperative data of the studied population.

**Table 4:** Follow-up postoperative parameters of the MTCs

Calcitonin (pg/ml)	< 1	100 (15.4%)
	1–10	315 (48.6%)
	> 10	233 (36%)
Post-op day of calcitonin measurement		43 (97)
CEA (ng/ml)	< 1	72 (15.8%)
	1–10	293 (64.4%)
	> 10	90 (19.8%)
Post-op day of CEA measurement		62 (124)
Suspicious/positive lymph nodes	Yes	117 (17.6%)
	No	546 (82.4%)
Calcitonin doubling time (months)		9 (16)
CEA doubling time (months)		8 (12)
Structural doubling time (months)		0 (9)
Death	Yes	41 (6.1%)
	No	630 (93.9%)
Survival (months)		53 (67)

### Correlations between lymph nodal disease in MCTs

For the correlations unknown stages (x) for T, N and M were excluded.

1. Correlations between lymph node disease and demographics

Concerning the correlation between age and lymph node stage, one can observe that there is no age difference between stages (p = 0.93).

Concerning lymph node stage and sex, we observe that N1a and N1b patient have higher probability of being male than female (p < 0.001). The male:female ratio for N0 stage is 0.54, for N1a is 0.59, and for N1b is 0.91.

Finally, there is no correlation between patients’ body mass index and lymph node stage (p = 0.2).

2. Correlations between lymph node disease and Preoperative characteristics of MTCs

Concerning preoperative biochemical and hematological data, all patients had no statistically significant difference between different lymph node stages for TSH, white blood cell count, lymphocyte count, platelets count, mean platelet volume, platelet distribution width, neutrophil-to-lymphocyte ratio and platelet-to-lymphocyte ratio (as displayed in Table 5).

**Table 5:** Correlations between lymph nodes stage and preoperative data

Variables	Lymph Node Stage (TNM)			Statistic, p value
Median (Interquartile range)	Ν0	Ν1a	Ν1b	
TSH (mIU/L)	1.76 (11.65)	1.88 (9.36)	1.81 (11.99)	χ^2^(2) = 0.922, p = 0.631
Calcitonin (pg/ml)	100 (50100)	235 (57245)	538 (154,488)	χ^2^(2) = 27.448, p < 0.001 N0-N1a*, N1a-N1b***
CEA (ng/ml)	11.58 (73,200)	13 (73300)	20.67 (128,500)	χ^2^(2) = 4.552, p = 0.103
Calcium (mg/dl)	9.3 (50.8)	9.24 (42.81)	8.8 (16.47)	χ^2^(2) = 9.881, p = 0.007 N0-N1b**, N1a-N1b*
WBC counts (K/L)	6835 (9390)	7120 (15600)	7200 (18000)	χ^2^(2) = 3.675, p = 0.159
Neutrophils (/μL)	4040 (55410)	4570 (66800)	4530 (78120)	χ^2^(2) = 10.392, p = 0.006 N0-N1b*
Lymphocytes (/μL)	2024 (43813)	2075.1 (1785360)	2140 (2654260)	χ^2^(2) = 1.597, p = 0.450
Monocytes (/μL)	299 (440233)	400 (452638)	428 (743386)	χ^2^(2) = 17.333, p < 0.001 N0-N1a*, N0-N1b**
Platelets (/μL)	243000 (513000)	263000 (383000)	254000 (414000)	χ^2^(2) = 2.477, p = 0.290
Mean Platelet Volume	9.65 (86.1)	9.4 (77)	9.7 (37.6)	χ^2^(2) = 0.174, p = 0.917
Platelet Distribution Width	16.4 (84.6)	16.45 (52.7)	24.65 (76.4)	χ^2^(2) = 2.505, p = 0.286
Neutrophil-to-lymphocyte ratio	1.98 (10.14)	2.02 (12.55)	2.18 (37.28)	χ^2^(2) = 2.591, p = 0.274
Platelet-to-lymphocyte ratio	118.53 (668.85)	130.26 (408.77)	113.64 (486.36)	χ^2^(2) = 3.575, p = 0.167
Lymphocyte-to-monocyte ratio	8.14 (100.26)	5.35 (142.79)	4.98 (1714.1)	χ^2^(2) = 8.176, p = 0.017 N0-N1a*

^*^p < 0.05, **p < 0.01, ***p < 0.001

As expected, the higher the pre-operative calcitonin levels are the more advanced the lymph node stage (p < 0.001). There is also statistically significant correlation between N1b lymph node stage and reduced neutrophils (p = 0.006), elevated monocyte (p < 0.001) and for N1a stage reduced lymphocyte-to-monocyte ratio (p = 0.017) (Table 5).

3. Correlations between lymph node disease and MTCs characteristics according to pathology reports

As far as it concerns the correlations between lymph node stage and MTC characteristics according to pathology report, there was no statistically significant results on the number of lymph nodes resected (p = 0.286), calcitonin staining (p = 0.522), CEA staining (p = 0.269), thyroglobulin staining (p = 0.084), CD 133 (p = 0.611), CD 44 (p = 0.48), HRAS (p = 0.814), KRAS (p = 0.898), NRAS (p = 0.487), Ki-67>5% (p = 0.726) and RET positivity (p = 0.077) as displayed in Table 6.

**Table 6:** Correlations between lymph node stage and tumor characteristics

Variables	Lymph Node Stage (TNM)			Statistic, p value
Median (Interquartile range) / N (%)	Ν0	Ν1a	Ν1b	
Number of lymph nodes resected	16 (85)	16 (53)	25 (76)	χ^2^(2) = 2.505, p = 0.286
Number of positive lymph nodes	0 (85)	1 (50)	3 (68)	χ^2^(2) = 102.960, p < 0.001 N0-N1a***, N0-N1b***
Lymph node ratio	0 (1)	0.17 (1)	0.21 (1.93)	χ^2^(2) = 106.384, p < 0.001 N0-N1a***, N0-N1b***
Diameter of tumor (mm)	15 (99.5)	18 (69)	20 (129)	χ^2^(2) = 23.765, p < 0.001 N1a-N1b*, N0-N1b***
*Uni-Multifocal*				
Unifocal	330 (80.7%)	118 (73.3%)	92 (70.2%)	χ^2^(2) = 7.784, p = 0.020
Multifocal	79 (19.3%)	43 (26.7%)	39 (29.8%)	
*Extrathyroidal extension*				
Yes	42 (10.2%)	27 (16.7%)	36 (27.9%)	χ^2^(2) = 24.511, p < 0.001
No	368 (89.8%)	135 (83.3%)	93 (72.1%)	
*Capsular invasion*				
Yes	53 (13%)	36 (22.6%)	51 (41.1%)	χ^2^(2) = 47.132, p < 0.001
No	354 (87%)	123 (77.4%)	73 (58.9%)	
*Calcitonin Staining*				
Negative	9 (2.6%)	1 (0.8%)	1 (1%)	χ^2^(6) = 5.173, p = 0.522
Mild	87 (25%)	34 (26.4%)	22 (21.8%)	
Moderate	38 (10.9%)	20 (15.5%)	11 (10.9%)	
Intense	214 (61.5%)	74 (57.4%)	67 (66.3%)	
*CEA Staining*				
Negative	27 (13.3%)	9 (14.3%)	9 (17.6%)	χ^2^(6) = 7.6, p = 0.269
Mild	47 (23.2%)	23 (36.5%)	12 (23.5%)	
Moderate	12 (5.9%)	5 (7.9%)	5 (9.8%)	
Intense	117 (57.6%)	26 (41.3%)	25 (49%)	
*Thyroglobulin staining*				
Negative	213 (89.1%)	71 (79.8%)	45 (88.2%)	χ^2^(6) = 11.133, p = 0.084
Mild	23 (9.6%)	16 (18%)	4 (7.8%)	
Moderate	2 (0.8%)	0 (0%)	0 (0%)	
Intense	1 (0.4%)	2 (2.2%)	2 (3.9%)	
*CD133 staining*				
Yes	32 (56.1%)	10 (45.5%)	5 (62.5%)	
No	25 (43.9%)	12 (54.5%)	3 (37.5%)	χ^2^(2) = 0.985, p = 0.611
*CD44 staining*				
Yes	30 (55.6%)	10 (50%)	6 (75%)	χ^2^(2) = 1.469, p = 0.480
No	24 (44.4%)	10 (50%)	2 (25%)	
*RET*				
Positive	24 (17.6%)	13 (21.3%)	19 (32.2%)	χ^2^(2) = 5.117, p = 0.077
Negative	112 (82.4%)	48 (78.7%)	40 (67.8%)	
*HRAS*				
Positive	2 (2.9%)	1 (2.9%)	1 (5.9%)	χ^2^(2) = 0.411, p = 0.814
Negative	68 (97.1%)	33 (97.1%)	16 (94.1%)	
*KRAS*				
Positive	3 (4.1%)	1 (2.9%)	1 (5.6%)	χ^2^(2) = 0.215, p = 0.898
Negative	70 (95.9%)	33 (97.1%)	17 (94.4%)	
*NRAS*				
Positive	2 (2.8%)	0 (0%)	0 (0%)	
Negative	70 (97.2%)	33 (100%)	18 (100%)	χ^2^(2) = 1.440, p = 0.487
*Ki-67 (> 5%)*				
Yes	55 (38.5%)	17 (38.6%)	16 (45.7%)	χ^2^(2) = 0.641, p = 0.726
No	88 (61.5%)	27 (61.4%)	19 (54.3%)	
*Lymphovascular invasion*				
Yes	74 (21%)	45 (36%)	56 (45.5%)	χ^2^(2) = 30.064, p < 0.001
No	278 (79%)	80 (64%)	67 (54.5%)	
*Desmoplasia*				
Yes	11 (4.7%)	4 (6.3%)	8 (12.9%)	
No	224 (95.3%)	60 (93.8%)	54 (87.1%)	χ^2^(2) = 5.562, p = 0.062

^*^p < 0.05, **p < 0.01, ***p < 0.001

As expected, there were more positive lymph nodes between stages (p < 0.001), as well as higher lymph node ratio (p < 0.001). Equally, logical seems the statistically significant correlation between lymph node stage and diameter of the tumor (p < 0.001). The large the tumor the more advance the lymph node stage is expected to be. Moreover, the multifocality of the MCTs is positively correlated (p = 0.02) to N1 stage (both N1a and N1b). As expected, more aggressive tumors -giving lymph node metastasis- are those with extrathyroidal extension (p < 0.001), capsular invasion (p < 0.001) and lymphovascular invasion (p < 0.001). Desmoplastic reaction seems to have a trend of correlation with lymph node metastasis but not statistically significant at the present study (p = 0.062). All the above are shown in Table 6.

As displayed in Table 7, there is statistically significant correlation between T and M stages (p < 0.001) with N stages. The more advanced the T stage, the higher the probability to give lymph node metastasis. Moreover, when distant metastasis is present it is high probable that there is also lymph node metastasis (in 97% of the cases).

**Table 7:** Correlations between lymph node stage and tumor and metastasis stages

Variables		Lymph Node Stage (TNM)			Statistic, p value
Median (Interquartile range) / N (%)		Ν0	Ν1a	Ν1b	
Tumor stage (TNM)	Τ1	272	95	56	p < 0.001
	Τ2	91	36	28	
	Τ3	36	26	32	
	Τ4	11	8	13	
Distant metastasis (TNM)	Μ0	397	153	119	p = 0.001
	Μ1	14	13	13

4. Correlations between lymph node disease and follow-up characteristics of MTCs

Surprisingly, there is no statistically significant difference between the median survival in months and the lymph node stage (p = 0.439). Moreover, postoperative CEA measurements seem to have a trend for correlation with lymph node stage (p = 0.058). On the other hand, all other parameters recorded in the present study were statistically significant correlated with lymph node staging. Analytically, more advance N stages have a higher probability of having postoperative calcitonin levels more than 10pg/ml (p < 0.001). Additionally, N1 stage has statistically significant correlation with postoperative suspicious/positive lymph nodes (p < 0.001). All post-operative correlation with lymph node stage is displayed in Table 8.

**Table 8:** Correlations between lymph nodes and follow-up postoperative parameters

Variables		Lymph Node Stage (TNM)			Statistic, p value
Median (Interquartile range) / N (%)		Ν0	Ν1a	Ν1b	
Calcitonin (pg/ml)	< 1	57 (16.3%)	14 (10.9%)	11 (8.9%)	χ^2^(4) = 37.805, p < 0.001
	1–10	198 (56.7%)	61 (47.7%)	42 (34.1%)	
	> 10	94 (26.9%)	53 (41.4%)	70 (56.9%)	
Post-op day of calcitonin measurement		35 (2919)	60 (2919)	60 (3649)	χ^2^(2) = 16.324, p < 0.001 N0-N1a**, N0-N1b**
CEA (ng/ml)	< 1	40 (17.2%)	18 (18.2%)	10 (10.6%)	χ^2^(4) = 9.122, p = 0.058
	1–10	157 (67.4%)	64 (64.6%)	57 (60.6%)	
	> 10	36 (15.5%)	17 (17.2%)	27 (28.7%)	
Post-op day of CEA measurement		60 (2920)	63 (3131)	85 (3649)	χ^2^(2) = 5.568, p = 0.062
Suspicious/positive lymph nodes	Yes	39 (11%)	29 (22.1%)	40 (33.1%)	χ^2^(2) = 31.862, p < 0.001
	No	314 (89%)	102 (77.9%)	81 (66.9%)	
Calcitonin doubling time (months)		9 (2161)	9 (9922)	12 (1020)	χ^2^(2) = 6.537, p = 0.038 N0-N1b*
CEA doubling time (months)		8 (1173)	6 (384)	7 (960)	χ^2^(2) = 0.731, p = 0.694
Structural doubling time (months)		0 (72)	0 (30)	5 (80)	χ^2^(2) = 8.066, p = 0.018 N1a-N1b*
Death	Yes	11 (3.2%)	14 (10.2%)	13 (10.4%)	χ^2^(2) = 12.995, p = 0.002
	No	336 (96.8%)	123 (89.8%)	112 (89.6%)	
Survival (months)		53 (134)	51 (131)	60 (180)	χ^2^(2) = 1.644, p = 0.439

^*^p < 0.05, **p < 0.01, ***p < 0.001

## Discussion

MTC is a rare disease, but it accounts for 13.4% of deaths related to thyroid cancer. The ATA and European Society of Medical Oncology (ESMO) guidelines on MTC report an excellent prognosis for stage I-III (100%-71%), which decreases in stage IV (21%) [2, 19]. Stage IV disease diminishes overall survival in the Danish population, while nodal and distant metastases are independent risk factors for disease progression [22]. The presence of lymph node metastases during the course of MTC increases the risk for distant metastases and worsens the disease prognosis [4, 6, 7, 22–24]. The extent of lymph node metastases is a critical prognostic factor, strongly associated with disease recurrence and overall survival rates[2]. Therefore, it is crucial to determine the risk factors that could predict nodal metastases in MTC in order to determine risk stratification and optimize the surgical approach.

Regarding demographic factors, age and male gender have been already established as predictive factors of disease prognosis. Older age has been correlated with worse overall and disease-free survival. Kotwal et al. suggested that age > 55 years is an independent factor for decreased overall survival in MTC [23] This is also supported by additional studies with different age cut-offs, suggesting 40, 45 and 50 years [6, 9, 24, 25]. Our study highlights that age has no impact on lymph node stage. Furthermore, male gender appears to correlate with a worse node stage than female gender. Costa et al. reported worse survival rates for male patients, even after adjusting for age and disease stage [8]. In the studies by Hassan et al. and Torresan et al., univariate analysis revealed male gender as a poor prognostic factor [4, 25]. A higher incidence of lateral cervical nodal metastasis has been found in male patients [26]. Our study confirmed that male gender is associated with advanced nodal stage.

Basal serum calcitonin and CEA levels are widely acknowledged factors that influence the surgical approach for MTC. The ATA and ESMO guidelines suggest different extents of lymph node dissection based on preoperative basal serum calcitonin [2, 19]. ESMO guidelines suggest strict basal serum calcitonin thresholds for different types of nodal dissection [19]. ATA guidelines propose total thyroidectomy with central compartment lymph node dissection if there is no evidence of nodal involvement. However, a consensus on basal calcitonin levels for lateral lymph node dissection has not been reached. Nevertheless, ipsilateral neck dissection is recommended in the case of nodal involvement, with contralateral neck dissection if basal calcitonin is > 200 pg/ml [2]. The surgical approach to cervical lymph nodes based on preoperative serum calcitonin levels remains debatable, as extensive surgical approaches are associated with higher complication rates, and the beneficial impact on disease prognosis is controversial. Several studies have attempted to assess the role of basal calcitonin and CEA levels in lymph node metastases. Bae et al. proposed cut-off levels of basal serum calcitonin for central and lateral compartment lymph node metastases, thus a basal calcitonin level of 226.6 pg/ml and 755 pg/ml suggests ipsilateral and contralateral central compartment metastases, while levels of 237 pg/ml and 296 pg/ml suggest ipsilateral and contralateral lateral metastases [1]. Zhang et al. also evaluated the role of preoperative calcitonin levels on poor prognosis and suggested that higher basal serum calcitonin levels correlate with higher TNM stage and poorer prognosis [27]. Furthermore, basal serum calcitonin levels > 309 pg/ml have been associated with disease recurrence [28]. Regarding CEA levels, higher preoperative levels lead to a higher incidence of lateral cervical compartment nodal metastases and worse survival outcomes [29, 30]. Preoperative CEA levels > 30ng/ml increase the risk for central compartment lymph node metastases [7]. Our study highlights the impact of preoperative basal serum calcitonin and CEA levels on disease progression, as they appear to be involved in nodal recurrence. It should be noted that the mean values of preoperative serum calcitonin levels that worsen disease prognosis with regard to nodal recurrence are 1416.81 pg/ml. In terms of lymph node metastases, the mean value of preoperative serum calcitonin and CEA levels in node-positive disease (N1a) are 235 pg/ml and 13 ng/ml, respectively, and in N1b disease, they are 538 pg/ml and 20.67 ng/ml. It should be mentioned that standard deviation is high, as seen in Table 5. Therefore, considering these results, it seems crucial to re-evaluate the surgical approach for prophylactic nodal dissections in MTC based on the established basal serum calcitonin level limits.

Pathological features for tumor aggressiveness of MTC have already established and widely adopted. Extrathyroidal tumor extension, capsular invasion, lymphovascular invasion, multifocality, and tumor size are essential factors that characterize tumor biological behavior and guide disease management. Several studies on MTC confirm the importance of these pathological factors on tumor aggressiveness. Gross extrathyroidal extension, tumor size, advanced T stage, tumor multifocality, thyroid capsule invasion, and lymphovascular invasion are risk factors for cervical lymph node metastasis and, therefore, poorer prognosis [4, 7, 23, 25, 26, 31, 32]. Fan et al. proposed a cut-off for tumor size of 1cm as a risk factor for nodal metastases [7]. Larger tumors > 1 cm have been found to metastasize more easily according to Aubert et al. [31]. Meng et al. suggested a cut-off point of 2 cm as critical for nodal metastases [9]. Our results align with these studies, as these pathological characteristics affect cervical lymph node metastases.

Furthermore, Ki-67 is a proliferation marker used in several malignancies to evaluate tumor biological behavior. A Ki-67 index > 3% is related to more aggressive tumor biology. Along with Ki-67, mitotic count (> 5/10 HPFs) and tumor necrosis are also considered. These markers also have been applied in MTC to assess tumor behavior. Combined Ki-67, mitotic count, and tumor necrosis could be strong predictors of overall survival [33]. Alzumaili et al. reported risk factors related to tumor aggressiveness, and, except for extrathyroidal extension, increasing tumor size, T stage, nodal metastasis and their largest size, high mitotic count (> 5/10HPFs), and tumor necrosis were also considered important predictors for worse disease-free survival, local recurrence-free survival, and distant metastasis-free survival [32]. RET mutation has also been evaluated in our study in sporadic cases and found to be related to nodal metastases. The RET variant has also been proposed as negative predictor of prognosis in the literature [25].

Stroma desmoplastic reaction around tumor has become a subject of interest as a novel marker of tumor biological behavior that could influence therapeutic strategy. This pathological factor has been already evaluated as possible marker since 2006 and has been supported as a reliable marker for nodal metastasis [13, 34]. Desmoplasia has recently revisited in the international literature, suggesting that a negative desmoplastic reaction could indicate a non-invasive MTC, potentially limiting surgical extent [10, 11, 35]. However, these studies had several limitations, and the issue is still debatable [36]. On the other hand, some studies proposed that the presence of desmoplastic reaction around tumor could be a negative prognostic factor [12]. Aubert et al. and Gomez- Ramirez et al. reported that stroma desmoplastic reaction enhances nodal metastases [12, 31]. Therefore, Machens et al. suggested that tumor desmoplasia may surpass basal serum calcitonin as a biomarker [37]. Regarding the results of this study, negative desmoplastic reaction has a higher possibility of node-negative disease, but nodal metastases cannot be excluded. We failed to prove that stromal desmoplastic reaction can be a statistically significant prognosticator of nodal metastasis (p = 0.062). Additionally, lateral cervical compartment nodal metastases were found to be related. Further prospective studies are mandatory to obtain reliable results about desmoplasia and its appliance in MTC.

Serum inflammation-based scores have been proposed as indicators of systemic inflammation caused by tumorigenesis and have prognostic value. Xu et al. also revealed that a higher neutrophil-to-lymphocyte ratio is related to poorer prognosis [18]. Figueiredo et al. failed to demonstrate that a higher neutrophil-to-lymphocyte ratio could predict lymph node metastases [17]. Li et al. also reported that a lymphocyte-to-monocyte ratio < 4.7 could indicate postoperative serum calcitonin progression [16]. According to Li et al., the platelet-to-lymphocyte ratio was predictive of capsular invasion, indicating aggressive tumor behavior [16]. Higher neutrophil-to-lymphocyte and platelet-to-lymphocyte ratios have been shown to be indicative of advanced disease [17]. In our study, only the lymphocyte-to-monocyte ratio is decreased in cases with N1b nodal involvement.

We should also point out the limitations of our study. First of all, the retrospective design of data collection makes our study vulnerable to selection bias. Due to the rarity of MTC and the differences in clinical practice over the decades, a 10-year retrospective data collection period was proposed. Furthermore, a significant amount of missing data on confounding factors weakens the evidence of our study. However, given the rarity of this malignancy, even 50% of missing data could provide us with trends in the evaluation of distinct factors. Racial differences were not considered in this multicenter study, as diagnostic and therapeutic strategies for MTC are universally accepted. Despite these limitations, it should be mentioned that this is the largest multicenter 10-year retrospective study on predictive factors for tumor aggressiveness, that could shed light on the management of MTC.

## Conclusion

MTC is the third most common thyroid malignancy and represents an aggressive type of thyroid cancer. Therefore, it is essential to identify key factors that enhance tumor aggressiveness to develop new diagnostic and therapeutic strategies. Our study features the biochemical and pathological factors associated with tumor aggressiveness and proposes new factors that should be taken into consideration. Preoperative inflammatory ratios, tumor desmoplastic reaction, and lymph node ratio could be valuable in managing MTC. Further prospective studies should be conducted to assess the efficacy of these factors and to establish new diagnostic and therapeutic options.
